# Supplementary material for: Solid-State Stability Profiling of Ramipril to Optimize Its Quality Efficiency and Safety
Source: Pharmaceutics. 2021 Oct 2;13(10):1600. doi: 10.3390/pharmaceutics13101600 (PMC8538641; doi:10.3390/pharmaceutics13101600)
Supplement: Supplementary file 1 [file pharmaceutics-13-01600-s001.zip › pharmaceutics-1326261-SI-done.pdf]

pharmaceutics

MDPI

Supplementary materials: Solid-State Stability Profiling of Ramipril to Optimize Its Quality Efficiency and Safety

Katarzyna Regulska, Joanna Musiał and Beata J. Stanisz

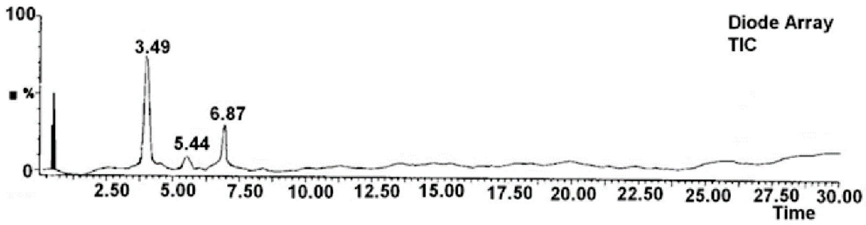

Figure S1. HPLC-MS chromatogram for degraded RAM in tablets (RH 76.0%; T = 318 K).

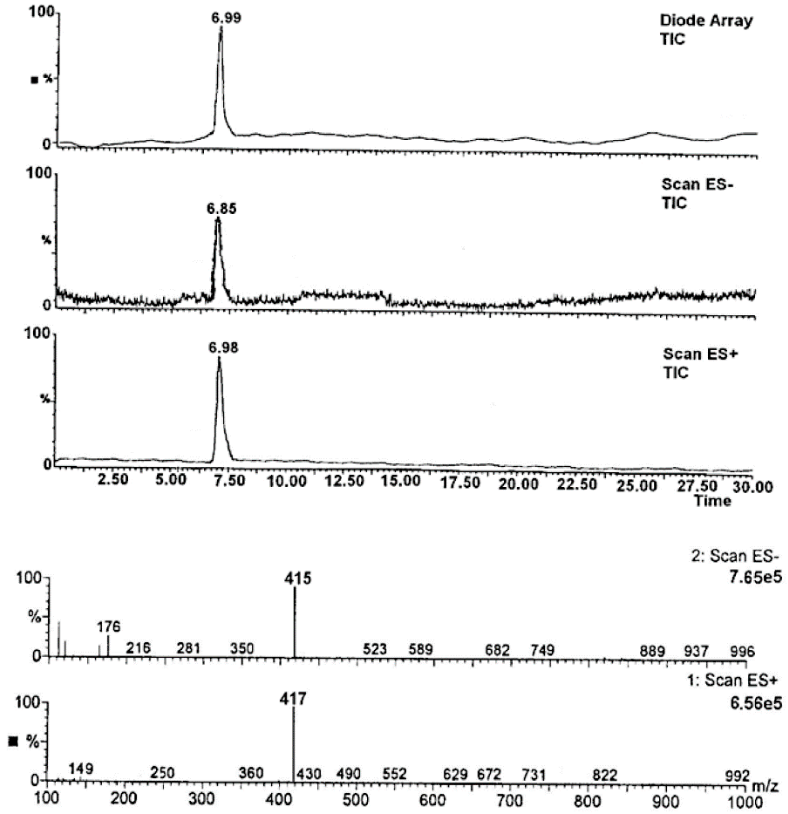

Figure S2. HPLC-MS chromatogram and mass spectrum for solid state undegraded RAM sample.
